# Potential Role of Pain Catastrophic Thinking in Comorbidity Patients of Depression and Chronic Pain

**DOI:** 10.3389/fpsyt.2022.839173

**Published:** 2022-07-08

**Authors:** Yuanyuan Chen, Peijun Ju, Qingrong Xia, Peng Cheng, Jianliang Gao, Loufeng Zhang, Hua Gao, Xialong Cheng, Tao Yu, Junwei Yan, Qiru Wang, Cuizhen Zhu, Xulai Zhang

**Affiliations:** ^1^Anhui Clinical Research Center for Mental Disorders, Affiliated Psychological Hospital of Anhui Medical University, Hefei, China; ^2^Anhui Clinical Center for Mental and Psychological Diseases, Hefei Fourth People's Hospital, Hefei, China; ^3^Department of Geriatric Psychology, Anhui Mental Health Center, Hefei, China; ^4^Shanghai Mental Health Center, Shanghai Jiao Tong University School of Medicine, Shanghai, China; ^5^Shanghai Key Laboratory of Psychotic Disorders, Shanghai, China; ^6^Minhang Branch, Department of Pharmacy, Fudan University Shanghai Cancer Center, Shanghai, China

**Keywords:** depression, chronic pain, comorbidity, pain catastrophizing, pain-related anxiety

## Abstract

**Background:**

Although comorbidity of major depressive disorder (MDD) and chronic pain (CP) has been well-studied, their association with pain catastrophizing is largely elusive. This study aimed to investigate the potential effects of pain catastrophizing in patients with a comorbidity.

**Methods:**

In total, 140 participants were included in this study and divided into three groups according to the Diagnostic and Statistical Manual of Mental Disorders and the International Association for the study of pain (i.e., the comorbidity group: patients with depression with chronic pain, *n* = 45; depression group: patients with depression without chronic pain, *n* = 47; and healthy controls: *n* = 48). The Hamilton Depression Rating Scale (HAMD)-24 and Hamilton Anxiety Rating Scale (HAMA)-14 were used by professional psychiatrists to evaluate the severity of depression and anxiety. Beck Depression Inventory-II (BDI-II) and Beck Anxiety Inventory (BAI) were conducted by patients' self-report to assess the symptom severity. The pain intensity numerical rating scale (PI-NRS) was used to assess the pain intensity. Pain Catastrophizing Scale (PCS) and Pain Anxiety Symptoms Scale (PASS) were used to estimate pain-related negative thinking.

**Results:**

The results showed that PASS and PCS scores were significantly different among the three groups. Particularly, the scores in the comorbidity group were the highest. The Pearson correlation analysis revealed a positive correlation between PCS (including the patients' helplessness, magnification, rumination, and total scores) and the severity of depression symptoms, anxiety symptoms, and pain intensity (*P* < 0.05). A stepwise regression analysis further demonstrated that the total PCS score, high monthly income level, and BDI score had positive impacts on PASS (*P* < 0.05). We also found that the total BDI score, disease course ≥1 year, and pain intensity had positive effects on PCS (*P* < 0.05), whereas years of education (≤ 12 years) had a negative effect on PCS (*P* = 0.012). In all, we have clearly demonstrated that PCS and PASS could serve as potentially predictive factors in patients suffering from comorbidity of MDD and CP.

**Conclusion:**

Our results suggested that the pain-related catastrophic thinking and anxiety were more severe in the comorbidity group than in MDD-only group and healthy group. Pain-related catastrophizing thoughts and anxiety may have potentially effects on the comorbidity of depression and chronic pain.

## Introduction

Empirical research has shown a strong correlation between major depressive disorder (MDD) and chronic pain (CP). Epidemiological studies have explored that the average prevalence of pain in MDD is as high as 65%, and the average prevalence of MDD in patients with CP is 52% (1, 2); the mean percentage of MDD is double in patients with CP compared with those without CP (3–5). Previous studies have identified that there is a significant relationship between lifetime prevalence of MDD and CP incidents, and those patients with the comorbidity of MDD and CP would more likely experience worse outcomes comparing with either one of them alone. High proportion of disease relapse and disability would lead to substantial impairment in physical, occupational, and social functioning in the comorbidity of MDD and CP (6–8). Multiple studies have demonstrated that MDD contributes to more intense pain and greater pain-related disability (1, 9). Although the interaction of physical and psychological factors are obvious and indisputable features of this comorbidity (10), few studies have clarified the underlying mechanism (11). Thus, to seek new evaluation strategies and improve the clinical treatment effect, it is necessary to perform a comprehensive study on the comorbidity of MDD and CP.

Psychosocial factors, such as pain-related anxiety and catastrophic thoughts, are critical determinants of differences in the development of MDD or CP (12, 13). Pain catastrophizing involves exaggerated adverse cognition in response to ongoing, anticipated, or recalled pain and associates with increased anxious emotional responses and heightened pain intensity. The detrimental role of pain catastrophizing and pain anxious feeling is well-documented and is widely acknowledged as a key predictor of coping with pain (14). For instance, several observational studies have conceptualized catastrophic thoughts to explain the exaggerated negative perceptions and avoidance behavior of patients with comorbidity (15, 16). A substantial body of evidence suggests that a depression-relevant scale (17, 18), anxiety-related scale, and catastrophic thought scale (19–21) are promising assessment instruments to detect negative feelings in patients with CP or MDD, respectively (22). These scales hold superior prognostic value for CP or MDD incidence and outcomes compared with many other variables (15). Thus, they are widely used in experimental and clinical research. At present, evidence-based guidance for psychopathological approaches that predict vulnerability risk factors for these comorbidity patients of MDD and CP is lacking; a guideline may help to understand how and why some individuals with comorbidity diseases have an underlying latent mental disorder (23).

From a clinical perspective, the majority of studies addressing the relationship between MDD and CP have limitations (17). First, most studies in this field were likely biased as the comparison was carried out between patients with MDD comorbid CP with healthy subjects; evidence from MDD patients with CP or without CP is lacking. Second, ample research has focused on patients' symptoms with fluctuations in depression-related and pain-related severity, which may assess symptom-dependent negative effects but not socially or psychologically related forms of comorbidity of MDD and CP. Thus, contextual features of pain-related fearful interpretations, avoidance behaviors, physiological responses, and cognitive interference in patients with comorbidity of MDD and CP need to be assessed multidimensionally (10, 24). Third, studies regarding anxiety-dependent stimulus features on catastrophic thoughts might point to new avenues of intervention strategy for comorbidity symptoms. In light of these information, we hypothesized that pain catastrophizing would exaggerate anxious emotion and perceptions of pain intensity and act as a key mediating role in the comorbidity of MDD and CP. This study was based on the classical MDD and CP disease model for better understanding of the association between the comorbidity symptoms and the psychosocial factors.

## Materials and Methods

### Participants

**Figure 1:**
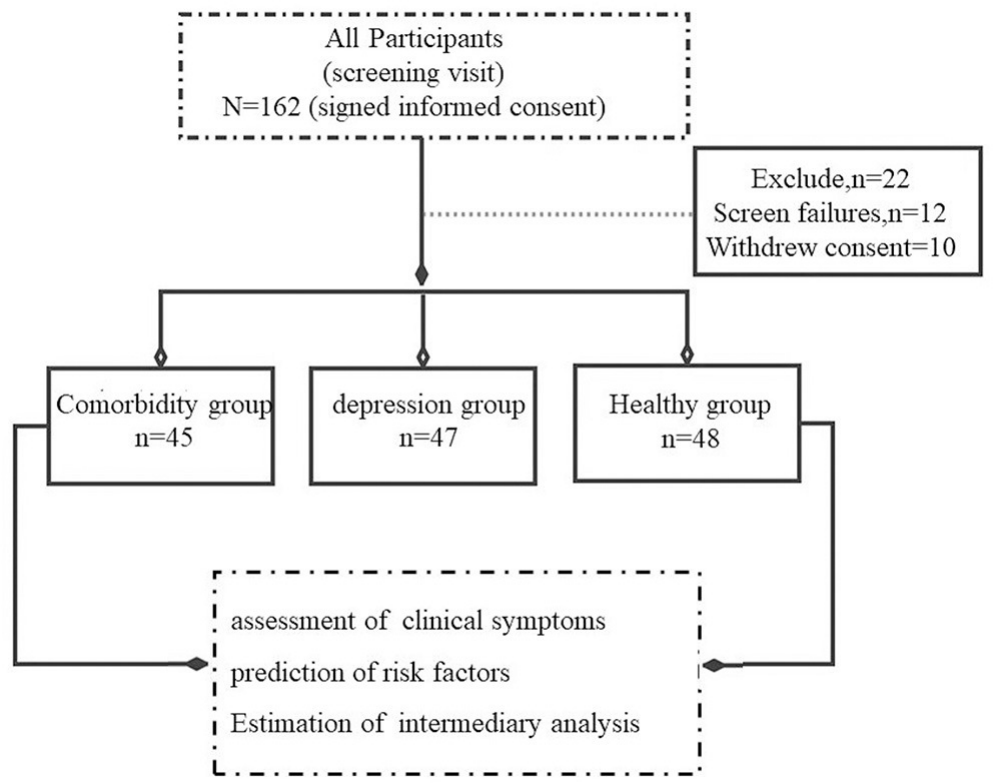
Study selection process.

This cross-sectional cohort study was conducted at the Anhui Mental Health Center (AMHC) between January 2019 and July 2021. According to trial standards, two professional doctors used the Mini-International Neuropsychiatric Interview (MINI) 6.0.0. to evaluate all participants. A total of 162 participants were initially evaluated, among which 12 participants could not complete the scale assessment and 10 individuals refused to sign the informed consent. Hence, 22 subjects who did not meet the experimental criteria were excluded from this experiment, and the remaining 140 participants were included. Ultimately, 140 participants were included in this study and divided into the following three groups according to the fifth edition of the Diagnostic and Statistical Manual of Mental Disorders (DSM-5), namely, comorbidity group (patients with MDD and CP, *n* = 45), depression group (depression patients without CP, *n* = 47), and control group (healthy controls, *n* = 48). The International Association for the Study of Pain (IASP) defines the CP as an unpleasant sensory and emotional experience, when it lasts or recurs for longer than 3 months (25). Participants in the comorbidity and depression groups were patients from AMHC, and patients in the control group were healthy people recruited from the hospital's physical examination center (Figure 1). The study was approved by the Medical Ethics Committee of AMHC. All participants provided written consent prior to study participation in accordance with the principles of the Declaration of Helsinki. The trial clinical registration number was chiCTR2000029917.

Patients in the comorbidity group met the following inclusion criteria: (1) fulfillment of the DSM-5 criteria and IASP for patients with depression and CP by two independent experienced psychiatrists; (2) aged between 16 and 60 years; (3) CP is not caused by physical trauma injury or serious somatic disease or inflammatory disease; (4) pain intensity numerical rating scale (PI-NRS) ≥3. The inclusion criteria for the depression group were as follows: (1) fulfillment of the DSM-5 criteria for depression by two independent experienced psychiatrists; (2) aged between 16 and 60 years; and (3) PI-NRS <3. All the participants did not have any of the following exclusion criteria: (1) history of craniocerebral trauma or somatic trauma; (2) history of severe inflammatory diseases, neurological diseases, or tumor-related diseases; (3) history of alcohol or other substance use or other mental disorders; (4) history of diabetes, hypertension, and endocrine disease; (5) pregnant or lactating women; and (6) administration of electroconvulsive therapy without convulsions 3 months before enrollment.

### Assessments

#### MINI 6.0.0

MINI 6.0.0, which is a concise diagnostic interview for psychiatric disorders used by psychiatrists in the United States and Europe, was used to verify the preliminary clinical diagnosis. All patients underwent MINI to confirm the clinical diagnoses of MDD with CP and MDD without CP (26).

### Demographic Characteristics

A self-reported questionnaire was used to collect information concerning age, gender, marital status, income status, employment status, educational level attained, duration of disease course, and health service accessibility.

### Hamilton Depression Rating Scale-24

Hamilton Depression Rating Scale-24 (HDRS-24) is the most commonly used depression scale worldwide because it has a high specificity to assess the severity of depression symptoms. The Cronbach's α score of HAMD-24 is 0.88, and the κ-score is 0.92. The HAMD-24 score may be used to define clinically relevant symptom levels as follows: <8 = no depression, 8–19 = mild depression, 20–34 = moderate depression and ≥35 = severe depression (2).

### Hamilton Anxiety Rating Scale-14

Hamilton Anxiety Rating Scale-14 was published more than 50 years ago as one of the first reliable and valid instruments that assess anxiety severity, and it has become the standard in the field. HAMA-14 was rated from 0 to 4 with general guidelines provided for distinguishing stage-wise anxiety severity. The items incorporate somatic and autonomic symptoms, respiratory and other physical tension, and emotional anxiety, such as fear and worry (27).

### Beck Depression Inventory-II

Beck Depression Inventory-II is a 21-item self-report questionnaire designed to assess the severity of depression symptoms. Each item is rated from 0 to 3 with general guidelines; its total score ranges from 0 to 63, and higher scores indicate more severe depressive symptoms. The psychometric properties of BDI-II have good internal consistency (Cronbach's alpha = 0.83) and excellent criterion validity (κ = 0.94) compared with other depression measures (17).

### Beck Anxiety Inventory

Beck Anxiety Inventory is a 21-item self-report instrument that assesses the grade of anxiety symptoms. Each item is rated from 0 to 3 with general guidelines; its total score ranges from 0 to 63, and higher scores indicate more severe anxiety symptoms. BAI has excellent internal consistency (Cronbach's alpha = 0.94) and good discriminant validity for anxiety disorders (28).

### Pain Catastrophizing Scale

Pain Catastrophizing Scale is a 13-item self-report instrument that assesses catastrophizing in the context of actual or anticipated pain. PCS measures catastrophizing as a multifaceted construct with three subscales, namely, rumination, magnification, and helplessness. PCS estimates how individuals respond to painful circumstances and indicates the degree to which they experience each thought or feeling when experiencing pain. Each item is rated from 0 (i.e., totally disagree) to 4 (i.e., totally agree) with general guidelines, its total score ranges from 0 to 52, and higher scores indicate more serious pain-related thoughts (15).

### Pain Anxiety Symptom Scale

Pain Anxiety Symptom Scale was translated into the Chinese version by Xiao-Yi Zhou. Chinese PASS is a 20-item questionnaire that comprises four subscales, namely, cognitive anxiety, escape/avoidance, fearful appraisal, and physiological anxiety. All items were rated on a scale ranging from 0 (i.e., never) to 5 (i.e., always). Its total score ranges from 0 to 100, and higher scores indicate more severe pain-related anxiety.

### Pain Intensity Numerical Rating Scale

Pain intensity numerical rating scale is a self-report questionnaire that assesses pain intensity and demonstrates strong construct validity and stability. In consideration of its simplicity and easy operation, it is widely used in the evaluation of clinical CP disease. This scale frequently measured on an 11-point pain intensity PI-NRS, where 0 indicates no pain and 10 indicates worst possible pain (29).

### Statistical Analysis

Statistical Package for the Social Sciences version 22.0 (IBM, Corp.) was used to analyze the data. The *t*-test, chi-squared test, and ANOVA were performed to compare the differences in continuous or categorical parameters among the three groups. Symptoms related to pain anxiety and pain catastrophizing were analyzed by repeated-measure ANOVA and Tukey *post-hoc* analyses. Pearson product-moment correlation coefficients were calculated to examine the relationships between severe degree of depression, pain, or anxiety and pain catastrophizing thoughts. A stepwise regression analysis was conducted to analyze the confirmatory factor analysis. *P* < 0.05 was considered statistically significant. Finally, the mediation analysis was performed to further test whether the relationship between the potentially risk factors and depressive symptoms was mediated by pain-related anxiety and pain catastrophizing thoughts. This analysis was carried out by quantifying the direct and indirect relationships among the independent variables, mediator (i.e., pain-related anxiety and pain catastrophizing thoughts), and dependent variable (i.e., depression symptoms). In the mediation models, all paths were reported as unstandardized ordinary least squares regression coefficients. A significance analysis was based on 5,000 bootstrap realizations, and a significant indirect effect was indicated when the bootstrap 95% confidence interval (CI) was not zero.

## Results

### Demographic, Depression, and Anxiety Psychometric Properties and Clinical Characteristics of the Three Groups

The demographic data, anxiety psychometric properties, and clinical characteristics of the three groups are shown in Table 1. A total of 140 participants were included in the final analyses. We recruited 45 patients (i.e., 29 females and 16 males) with MDD comorbid CP, 47 patients (i.e., 25 females and 22 males) with MDD but without CP, and 48 healthy controls (i.e., 20 females and 28 males). No remarkable differences in marital status, personal income, employment situation, years of education, and body mass index (BMI) were found among the three groups (*P* > 0.05). Psychometric properties and the clinical characteristics of depression and anxiety also showed no differences between the comorbidity and depression groups. However, pain intensity was more severe in the comorbidity group compared with the depression group (*P* = 0.025).

**Table 1:** Comparison of sociodemographic characteristics, anxiety, depression symptom, and pain intensity among the three groups.

	**Healthy group (*n* = 48)**	**Depression group (*n* = 47)**	**Comorbidity group (*n* = 45)**	***F/T/χ2***	***P***
Sex (*n*,%)
Female	20 (41.7)	25 (53.2)	29 (64.4)	4.839	0.089
Male	28 (58.3)	22 (46.8)	16 (35.6)		
Marital status (%)
No	21 (43.8)	25 (53.2)	22 (48.9)	0.850	0.654
Yes	27 (56.2)	22 (46.8)	23 (51.1)		
Monthly income (%)
≤ 5,000 yuan	32 (66.7)	26 (55.3)	30 (66.7)	1.722	0.423
>5,000 yuan	16(33.3)	21(44.7)	15 (33.3)		
Employment status (%)
No	15 (31.3)	20 (42.6)	13 (28.9)	2.204	0.332
Yes	33 (68.7)	27 (57.4)	32 (71.1)		
Years of education (%)
≤ 12 years	27 (56.2)	17 (36.2)	25 (55.6)	4.874	0.087
>12 years	21 (43.8)	30 (63.8)	20 (44.4)		
Course of disease (%)
<1 year	/	14 (29.8)	12 (26.7)	0.110	0.740
≥1 years	/	33 (70.2)	33 (73.3)		
BMI (kg/m^2^)	23.24 ± 4.38	21.90 ± 4.11	22.16 ± 3.32	1.522	0.222
Age	32.52 ± 10.54	33.55 ± 12.39	36.04 ± 13.26	1.035	0.358
HAMD-24	/	25.23 ± 11.20	30.44 ± 9.41	0.642	0.425
BDI	/	21.38 ± 10.43	28.02 ± 11.94	1.667	0.200
HAMA-14	/	13.00 ± 8.11	18.71 ± 8.85	1.745	0.190
BAI	/	33.38 ± 11.63	43.22 ± 12.04	1.211	0.274
PI-NRS		2.23 ± 2.28	5.58 ± 1.78	5.205	0.025

*BMI, body mass index; HAMD-24, Hamilton Depression Rating Scale-24; HAMA-14, Hamilton Anxiety Rating Scale-14; BDI, Beck Depression Inventory; BAI, Beck Anxiety Inventory; PI-NRS, pain intensity numerical rating scale*.

### Clinical Characteristics of Pain-Related Anxiety and Pain Catastrophizing Among the Three Groups

Pain Anxiety Symptoms Scale and PCS scales were used to evaluate the pain-specific anxiety and pain-related negative physiological cognitive responses of the patients. Four subscales of PASS were contained, namely, cognitive anxiety, escape/avoidance, fear of pain, and physiological anxiety. The results showed that total PASS score, fear of pain score, and physiological anxiety score were distinct among the three groups (*F* = 21.29, *P* < 0.001; *F* = 26.62, *P* < 0.001; *F* = 14.46, *P* < 0.001, respectively). The comorbidity group had significantly higher total PASS scores, fear of pain score, and physiological anxiety score (all *P* < 0.05, respectively) than the MDD and healthy groups. The three factors in the PCS scale, namely, magnification, rumination, and helplessness, were also assessed. Total PCS score, magnification score, rumination score, and helplessness score were significantly different among the three groups (*F* = 55.05, *P* < 0.001; *F* = 40.89, *P* < 0.001; *F* = 33.18, *P* < 0.001; *F* = 60.47, *P* < 0.001, respectively). Together, these results (Table 2) indicate that the comorbidity group is prone to anxiety-related pain and catastrophizing thoughts than the healthy and MDD groups.

**Table 2:** Pain-related anxiety and pain catastrophizing properties among three groups.

	**A Group**	**B Group**	**C Group**	***F***	***p***	***A* vs. *B***	***A* vs. *C***	***B* vs. *C***
	**(*n* = 48)**	**(*n* = 47)**	**(*n* = 45)**					
PASS total	26.83 ± 15.79	41.06 ± 19.73	50.27 ± 16.68	21.288	<0.001	<0.001	<0.001	0.013
Cognitive anxiety	7.38± 4.88	12.47 ± 6.66	14.38 ± 4.90	19.982	<0.001	<0.001	<0.001	0.101
Escape/avoidance	9.50 ± 5.95	11.36 ± 5.38	12.82 ± 5.14	4.259	0.016	0.102	0.004	0.206
Fear of pain	3.94 ± 3.34	8.19 ± 5.85	11.64 ± 5.79	26.617	<0.001	<0.001	<0.001	0.001
Physiologic anxiety	6.02 ± 4.52	9.04 ± 4.96	11.42 ± 5.09	14.463	<0.001	0.003	<0.001	0.020
PCS total	10.04 ± 8.35	21.94 ± 12.10	32.13 ± 9.70	55.054	<0.001	<0.001	<0.001	<0.001
Magnification	2.48 ± 2.18	5.17 ± 3.02	7.47 ± 2.74	40.893	<0.001	<0.001	<0.001	<0.001
Rumination	3.98 ± 3.63	7.55 ± 4.22	10.22 ± 3.20	33.184	<0.001	<0.001	<0.001	0.001
Helplessness	3.58 ± 3.30	9.21 ± 5.78	14.44 ± 4.91	60.469	<0.001	<0.001	<0.001	<0.001

*PASS, Pain Anxiety Symptoms Scale; PCS, Pain Catastrophizing Scale. A group, healthy group; B group, depression group; C, comorbidity group*.

### Factors Correlated With Pain-Related Anxiety and Pain Catastrophizing Thoughts

Remarkable correlations were found between the components of pain-related anxiety and depression symptoms. Specifically, self-reported depression symptoms had positive correlations with fear of pain, physiologic anxiety, and total PASS score (*r* = 0.472, *P* < 0.001; *r* = 0.394, *P* < 0.001; *r* = 0.432, *P* < 0.001). Meanwhile, self-reported anxiety symptoms had positive correlations with fear of pain, physiological anxiety, and total PASS score (*r* = 0.460, *P* < 0.001; *r* = 0.395, *P* < 0.001; *r* = 0.411, *P* < 0.001). Interestingly, from the perspective of professional psychiatrists, depression symptoms only had a positive relationship with physiologic anxiety (*r* = 0.243, *P* = 0.020), and somatic anxiety symptoms had a positive relationship with fear of pain (*r* = 0.219, *p* = 0.036). In addition, we found that the course of disease positively correlated with fear of pain and the total PASS score (*r* = 0.233, *P* = 0.025; *r* = 0.237, *P* = 0.023). On the contrary, we discovered that years of education and monthly income negatively correlated with fear of pain (*r* = −0.218, *P* = 0.036; *r* = −0.221, *p* = 0.034). Except for the above, we also found the pain intensity had positive related to the fear of pain and physiologic anxiety (*r* = 0.222, *P* = 0.033; *r* = 0.291, *p* = 0.005).

We evaluated the relationship between pain catastrophizing thoughts and depression symptoms. Self-reported depression symptoms had positive correlations with the helplessness score, magnification score, rumination score, and total PCS score (*r* = 0.499, *P* < 0.001; *r* = 0.362, *P* < 0.001; *r* = 0.242, *P* = 0.020; *r* = 0.423, *P* < 0.001). Similar to the previous results, self-reported anxiety symptoms had positive correlations with the helplessness, magnification, rumination, and total PCS scores (*r* = 0.433, *P* < 0.001; *r* = 0.433, *P* < 0.001; *r* = 0.320, *P* = 0.002; *r* = 0.435, *P* < 0.001). In addition, depression symptoms were also positively associated with the helplessness score, magnification score, rumination score, and total PCS score (*r* = 0.279, *P* = 0.007; *r* = 0.262, *P* = 0.012; *r* = 0.262, *P* = 0.012; *r* = 0.287, *P* = 0.006). Psychological anxiety and somatic anxiety had positive relations with the helplessness score (*r* = 0.238, *P* = 0.023; *r* = 0.227, *P* = 0.029). Furthermore, the pain intensity also had a strong correlation with helplessness, magnification, rumination, and total of PCS scores (*r* = 0.326, *P* = 0.002; *r* = 0.253, *P* = 0.015; *r* = 0.209, *P* = 0.045; *r* = 0.292, *P* = 0.005). Above-mentioned results are shown in Tables 3, 4.

**Table 3:** Factors correlation with pain-related anxieties.

**Factors**	**Pain Related Anxiety Scale**					
	**Fear of pain**		**Physiologic anxiety**		**PASS total**	
	***r***	***p***	***r***	***p***	***r***	***p***
Sex	−0.174	0.098	−0.252*	0.015	−0.215*	0.040
Course of disease	0.233*	0.025	0.193	0.065	0.237*	0.023
Years of education	−0.218*	0.036	−0.014	0.898	−0.059	0.575
Monthly income	−0.221*	0.034	0.016	0.880	−0.052	0.620
BDI	0.472**	<0.001	0.394**	<0.001	0.432**	<0.001
BAI	0.460**	<0.001	0.395**	<0.001*	0.411**	<0.001
HAMD-24	0.194	0.064	0.243*	0.020	0.183	0.081
HAMA-14	0.205	0.050	0.085	0.419	0.086	0.413
Psychological anxiety	0.193	0.066	0.121	0.251	0.076	0.471
Somatic anxiety	0.219*	0.036	0.064	0.542	0.122	0.247
PI-NRS	0.222*	0.033	0.291**	0.005	0.196	0.061

BDI, Beck Depression Inventory; BAI, Beck Anxiety Inventory; HAMD-24, Hamilton Depression Rating Scale-24; HAMA-14, Hamilton Anxiety Rating Scale-14; PI-NRS, pain intensity numerical rating scale. *P ≤ 0.05 and **P ≤ 0.001.

**Table 4:** Factors correlation with pain catastrophizing thoughts.

**Factors**	**Pain catastrophizing thoughts**							
	**Helplessness**		**Magnification**		**Rumination**		**PCS Total**	
	***r***	***p***	***r***	***p***	***r***	***p***	***r***	***p***
Course of disease	0.389**	<0.001*	0.324**	0.002	0.341**	0.001	0.398**	<0.001*
Years of education	−0.277**	0.008	−0.251*	0.016	−0.216*	0.039	−0.285**	0.006
Monthly Income	−0.306**	0.003	−0.234*	0.025	−0.205*	0.049	−0.291**	0.005
BDI	0.499**	<0.001*	0.362**	<0.001*	0.242*	0.020	0.423**	<0.001*
BAI	0.433**	<0.001*	0.433**	<0.001*	0.320**	0.002	0.435**	<0.001*
HAMD-24	0.279**	0.007	0.262*	0.012	0.262*	0.012	0.287**	0.006
HAMA-14	0.240*	0.021	0.179	0.088	0.126	0.231	0.208*	0.046
Psychological anxiety	0.238*	0.023	0.193	0.066	0.121	0.250	0.216*	0.038
Somatic anxiety	0.227*	0.029	0.153	0.147	0.126	0.230	0.189	0.072
PI-NRS	0.326**	0.002	0.253*	0.015	0.209*	0.045	0.292**	0.005

BMI, body mass index; HAMD-24, Hamilton Depression Rating Scale-24; HAMA-14, Hamilton Anxiety Rating Scale-14; BDI, Beck Depression Inventory; BAI, Beck Anxiety Inventory; PCS, Pain Catastrophizing Scale; PI-NRS, pain intensity numerical rating scale. *p ≤ 0.05 and **p ≤ 0.001.

### Independent Factors of Pain-Related Anxiety and Pain Catastrophizing Thoughts

**Figure 2:**
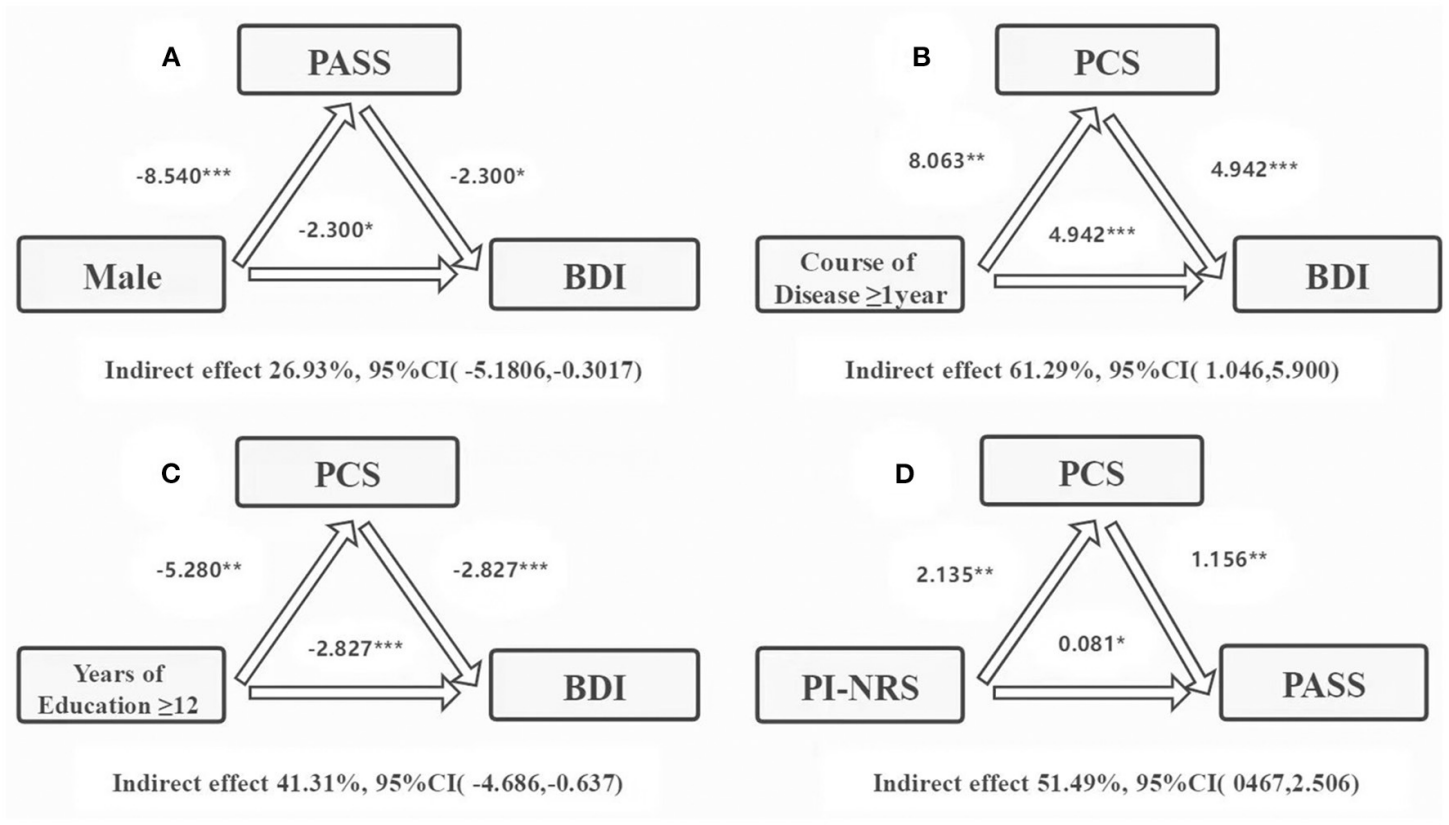
Mediation analysis of the role of pain-related anxieties and pain catastrophizing thoughts in mediating the relationship between male, course of disease ≥ 1 year, years of education ≥12, depressive symptoms, and pain intensity. PASS, Pain Anxiety Symptoms Scale; PCS, Pain Catastrophizing Scale; PI-NRS, pain intensity numerical rating scale; * *P* ≤ 0.05; ** *P* ≤ 0.01; *** *P* ≤ 0.001.

Stepwise regression analyses were performed to clarify the distinct role of pain-related anxiety and pain catastrophizing thoughts in the comorbidity of depression and CP. As shown in Table 5, the final model from forward regression indicated that the remarkable explanatory variables accounted for 60.5% of the variance in total PASS scores. A significant positive effect was found between PASS with the total PCS score, high monthly income level, and BDI score (β = 0.678, *t* = 9.025, *P* < 0.001; β = 0.194, *t* = *2.719, P* = 0.008; β = 0.204, *t* = 2.662, *P* = 0.009), whereas a significant negative impact was found between men and PASS score (β = −*0.169, t* = −*2.447, P* = 0.016). Furthermore, the regression model accounted for 53.1% of the variation in total PCS scores. A positive effect was explored between PCS with total BDI score, disease course ≥ 1 year, and pain intensity (β = 0.261, *t* = 2.646, *P* = 0.010; β = 0.291, *t* = 3.024, *P* = 0.003; β = 0.156, *t* = 2.098, *P* = 0.039), and a negative relationship was found between education >12 years and PCS score (β = –0.240, *t* = −2.562, *P* = 0.012). Finally, an examination of the mediator pathways revealed the indirect role of pain-related anxiety, pain catastrophizing thoughts, and pain intensity in depression symptom. Boot strapping results indicated that the indirect effect was significant (*P* < 0.05). The 95% CI did not contain zero, confirming the significant mediating effect of pain-related anxiety and pain catastrophizing thoughts for depression symptoms prediction (Figure 2).

**Table 5:** Prediction of risk factors by regression analysis.

**Dependent variables^**a**^**	**Independent variables**	***B***	**SE**	**Beta (β)**	***T***	***P***	***R^**2**^***
PASS	PCS Total	1.055	0.117	0.678	9.025	<0.001	0.605
	Sex^b^	−6.393	2.613	−0.169	−2.447	0.016	
	Monthly Income^c^	7.422	2.730	0.194	2.719	0.008	
	BDI Total^d^	0.330	0.124	0.204	2.662	0.009	
PCS	BDI Total	0.271	0.103	0.261	2.646	0.010	0.531
	Course of disease^e^	7.762	2.567	0.291	3.024	0.003	
	Course of disease^e^	7.762	2.567	0.291	3.024	0.003	
	Years of education^f^	−5.795	2.262	−0.240	−2.562	0.012	
	PI-NRS	0.712	0.339	0.156	2.098	0.039	

a *Dependent variables: PASS, Pain Anxiety Symptoms Scale; PCS, Pain Catastrophizing Scale; PI-NRS, pain intensity numerical rating scale*.

b *Sex; 1, female; 2, male*.

c *Monthly income: 1, ≤ 5,000 yuan; 2, >5,000 yuan*.

d *Beck Depression Inventory*.

e *Course of disease; 1, < 1 year; 2, ≥1 years*

f *Level of education; 1, ≤ 12 years; 2, >12 years. Significant level at P < 0.05*.

*B, unstandardized coefficient; SE, standard error; beta, standardized coefficient; R^2^, R square*.

## Discussion

The primary aim of this study was to investigate the potentially role of pain-related anxiety and pain catastrophizing in the patients with the comorbidity of MDD and CP. The results of this study verified our hypotheses that patients with comorbidity of MDD and CP could more easily slip back into negative psychological cognition, namely, pain-related anxiety and pain catastrophizing thoughts than patients with depression who had no pain symptoms. These findings also found that the sex, disease course, years of education, and pain intensity are the risk factors associated with disease severity. Pain-related anxiety and pain catastrophizing could be the mediating factors to affect the depressive symptoms. The results suggested that these vulnerability psychosocial factors could be considered as potential risk factors for the comorbidity of MDD and CP, which we should consider in the future.

Consistent with previous studies, patients with the comorbidity of MDD and CP had more pain-related anxiety symptoms than patients in the depression and control groups (6, 30). Although previous studies on depression showed that pain-related anxiety can induce pain experience, they did not investigate its accuracy (31). Our study demonstrated that the total PASS score and fear of pain score were most severe in the comorbidity group (32, 33). This result is in line with previous studies showing that anxiety sensitivity and anxiety severity are correlated with actual and expected pain-related fear (34). However, the scores of two PASS subscales (i.e., cognitive anxiety and escape/avoidance) were not different in the comorbidity group and depression group. These results explained that the comorbidity patients were prone to immerse thoughts about fear of pain. It is well-known that pain catastrophizing involves exaggerated maladaptive cognition or emotions in response to ongoing, anticipated, or recalled pain (14). This study showed that pain catastrophizing scores vary in the three groups. Similarly, the total PCS score and PCS subscale score (i.e., magnification, rumination, and helplessness scores) were highest in the comorbidity group. The psychological process of painful catastrophic thinking involves attention to pain, pain processing, and emotional and behavioral reactions caused by pain (35). When individuals suffer physical and psychological impairment, they are apt to get into extreme and absolute judgment, produce errors in cognition, and experience negative emotions and behaviors, in other words, the development of complex symptoms is expected in patients with the comorbidity of MDD and CP (36). At present, less is known about the risk factors that influence the elicitation or activation of pain-related anxiety and pain catastrophic thoughts in patients with comorbidity of MDD and CP. From a clinical perspective, increased knowledge about the risk factors associated with pain-related anxiety and pain catastrophic thinking might point to new avenues of intervention for those comorbidity patients (15). Interestingly, this study demonstrated that both patients' self-reported depression and anxiety symptoms were strongly positively correlated with fear of pain, physiologic anxiety, and total scores of PASS and PCS. Nevertheless, a professional psychiatrist found that depression symptom had positive correlation with physiologic anxiety in PASS. In addition, only symptom of psychological and somatic anxiety had positive correlation to helplessness in PCS. These results suggested that there was a disaccord between the professional doctor's assessment and the patient's self-reported symptoms, which attracted more attention in the future. In addition, we found that the education level and monthly income had a negative correlation with fear of pain and pain catastrophic thoughts, i.e., patients with a high education level and high income are less likely to experience fear of pain and negative pain-related thoughts. Consistent with previous studies, we found a negative correlation between men with physiologic anxiety and total PASS score (37–39). Furthermore, we also demonstrated the positive correlation between disease course with fear of pain and PCS. Together, these results demonstrated that negative pain thoughts and pain duration can imply poorer outcome in patients with the comorbidity of MDD and CP (23, 40).

Notably, this study demonstrated that sex, disease course, years of education, monthly income, pain intensity, and symptoms of disease are strongly associated with PASS and PCS scores (41–43). Specifically, we found that the severity of PCS, sex, monthly income, and severity of depression are potential effects on the PASS. Meanwhile, PASS had an indirect mediating effect between male sex and patients' self-reported depression symptoms. Our results also presented that depression severity, disease course, and years of education and strength of pain have potential roles on the PCS. Particularly, the PCS score had an indirect mediating effect on disease courses ≥1 year, depression severity, and pain-related anxiety. Moreover, we found the pain intensity had an indirect mediating effect on the PCS. Our findings suggest that these susceptible factors may be important in the occurrence of MDD and comorbid CP. The multiple evaluation is based on the patients' self-reports and professional psychiatrist's assessments in a clinical setting, available as strategies to broaden our perspectives. As such, we recommend the use of a multidimensional model in approaching the comorbidity of depression and CP in future clinical research.

Our findings should be interpreted with caution because of several limitations. First, this design is a cross-sectional study, which requires long-term clinical observation to obtain a more accurate information on the causal relationship of disease risk factors. Second, the sample size in this clinical research is relatively small; thus, future studies should target larger-sized cohorts with the comorbidity of depression and CP. Despite these limitations, this study provides a new perspective on the association of pain-related anxiety and pain catastrophic thoughts in the comorbidity of depression and CP to some extent. One strength of our study is the use of a multidimensional model in approaching the comorbidity of MDD and CP in clinical research. The risk factors of pain-related anxiety and PCS score were based on the results from the evaluation of patients' self-reported symptoms and the assessment results of professional psychiatrists. This study provides a comprehensive comparison relevant to understanding the consistency and variation in the occurrence of the comorbidity of MDD and CP.

In summary, this study indicates that the comprehensive assessment of multidimensional clinical symptoms, pain-related anxiety, and pain catastrophic thoughts should be considered when assessing patients with MDD and CP. Several risk-mediating factors deserve our attention to evaluate clinical sensitivity to comorbid mental disorders and CP. All our findings provided new insights into the assessment of pain-related anxiety and pain catastrophic thoughts, and it is hoped that these risk factors will provide more effective predicted effect for the clinical diagnosis and treatment on comorbidity of MDD and CP.

## Data Availability Statement

The original contributions presented in the study are included in the article/supplementary material, further inquiries can be directed to the corresponding authors.

## Ethics Statement

The studies involving human participants were reviewed and approved by Medical Ethics Committee of Anhui Mental Health Center. Written informed consent to participate in this study was provided by the participants' legal guardian/next of kin.

## Author Contributions

CZ and XZ were responsible for study design and manuscript editing. YC, PJ, and QX were responsible for literature searches, statistical analyses, and manuscript writing. PC, JG, LZ, HG, XC, TY, JY, and QW were responsible for clinical-scale assessment data collection. All authors contributed to the article and approved the submitted version.

## Funding

This study was supported by funding of Scientific and Technological Research Project of Anhui Provincial Science and Technology Department (201904a07020009), Hospital Project of Hefei Fourth People's Hospital (Grant Number: 2019023), Fund Project of Anhui Medical University (Grant Number: 2019xkj206), Shanghai Key Laboratory of Psychotic Disorders Open Grant (Grant Number: 13dz2260500), Natural Science Research Projects in Anhui Universities (Grant Number: KJ2020A0218), Applied Medicine Research Project of Hefei Health Committee (Grant Number: Hwk2020zd0016), Applied Medicine Research Project of Anhui Health Committee (Grant Number: AHWJ2021a036), and Natural Science Foundation of Minhang District (Grant Number: 2020MHZ063). The funding sources had no involvement in the study design, collection, analysis, and writing of this article and publication.

## Conflict of Interest

The authors declare that the research was conducted in the absence of any commercial or financial relationships that could be construed as a potential conflict of interest. The handling editor declared a past collaboration with one of the author's, CZ.

## Publisher's Note

All claims expressed in this article are solely those of the authors and do not necessarily represent those of their affiliated organizations, or those of the publisher, the editors and the reviewers. Any product that may be evaluated in this article, or claim that may be made by its manufacturer, is not guaranteed or endorsed by the publisher.

## Abbreviations

BAI: Beck Anxiety Inventory
BDI- II: Beck Depression Inventory-II
BMI: body mass index
CP: chronic pain
DSM-5: Diagnostic and Statistical Manual of Mental Disorders-5
HAMD-24: Hamilton Depression Rating Scale-24
HAMA-14: Hamilton Anxiety Rating Scale-14
IASP: International Association for the Study of Pain
MDD: major depressive disorder
MINI: Mini-International Neuropsychiatric Interview
PASS: Pain Anxiety Symptom Scale
PCS: Pain Catastrophizing Scale
PI-NRS: Pain intensity numerical rating scale.
